# The Role of Ablative Techniques in the Management of Hepatocellular Carcinoma: Indications and Outcomes

**DOI:** 10.3390/biomedicines11041062

**Published:** 2023-03-31

**Authors:** Asanda Koza, Ricky H. Bhogal, Nicos Fotiadis, Vasileios K. Mavroeidis

**Affiliations:** 1 Department of Interventional Radiology, The Royal Marsden NHS Foundation Trust, London SW3 6JJ, UK; 2 Department of Academic Surgery, The Royal Marsden NHS Foundation Trust, London SW3 6JJ, UK

**Keywords:** Hepatocellular carcinoma, ablative techniques, interventional radiology, radiofrequency ablation, microwave ablation, cryotherapy, irreversible electroporation

## Abstract

The management of hepatocellular carcinoma (HCC) remains complex and will continue to rely on the multidisciplinary input of hepatologists, surgeons, radiologists, oncologists and radiotherapists. With the appropriate staging of patients and selection of suitable treatments, the outcomes for HCC are improving. Surgical treatments encompassing both liver resection and orthotopic liver transplantation (OLT) are the definitive curative-intent options. However, patient suitability, as well as organ availability, pose essential limitations. Consequently, non-surgical options, such as ablative techniques, play an increasingly important role, especially in small HCCs, where overall and disease-free survival can be comparable to surgical resection. Ablative techniques are globally recommended in recognised classification systems, showing increasingly promising results. Recent technical refinements, as well as the emerging use of robotic assistance, may expand the treatment paradigm to achieve improved oncological results. At present, in very early stage and early stage unresectable disease, percutaneous thermal ablation is considered the treatment of choice. Owing to their different features, various ablative techniques, including radiofrequency ablation, microwave ablation, cryotherapy ablation and irreversible electroporation, have been shown to confer different comparative advantages and applicability. We herein review the role of available ablative techniques in the current complex multidisciplinary management of HCC, with a main focus on the indications and outcomes, and discuss future perspectives.

## 1. Introduction

Surgical treatments encompassing both liver resection and orthotopic liver transplantation (OLT) are the definitive curative-intent treatment options for hepatocellular carcinoma (HCC) [1]. There are, however, limitations to these surgical options primarily dependent upon patient suitability, as well as organ availability [2]. In addition, with limited grafts, emphasis and focus must be placed on indications for patient selection into appropriate treatment groups to minimise recurrence and achieve optimal outcomes. Hence non-surgical techniques, such as ablation, are an important treatment option, especially in small HCC, where ablation seems to have comparable overall and disease-free survival to surgical resection [3]. Ablation techniques are globally recommended in recognised classification systems, such as The Barcelona Clinic Liver Cancer classification (BCLC) and by societies including the European Society for the Study of the Liver (EASL) and the American Association for the Study of Liver Diseases (AASLD) [4]. With the increasing incidence of HCC, particularly in the context of non-alcoholic fatty liver disease (NAFLD), there is an increased focus on the optimal form of treatment for HCC occurring on the background of different aetiologies and the subsequent outcomes of the treatments [5]. As evidence emerges for effective treatment approaches, the management adjusts to reflect this with incorporation into the latest BCLC model. On review of the current literature, however, it remains clear that the management of HCC remains multifactorial and will continue to rely on input from the multidisciplinary mix of hepatologists, surgeons, radiologists, oncologists, radiotherapists and specialist nurses. Ablation techniques are showing promising results, and with the emerging use of robotic assistance, there is scope for furthering the treatment paradigm to achieve improved oncological results.

The purpose of this narrative review is to explore the role of ablative techniques in the management of HCC with a particular focus on the indications and outcomes of clinical studies from the past five years.

## 2. Hepatocellular Carcinoma

HCC is the sixth most common cancer and the third leading cause of cancer-related mortality worldwide [6]. It remains the most common primary malignancy of the liver, accounting for as much as 90% of liver cancer diagnoses [7]. It is of particular interest as, despite the advances made in screening, diagnosis and treatment, the incidence of HCC continues to rise worldwide, with an estimated 841,000 new cases in 2018 [7,8,9]. Late diagnosis of HCC in many cases has a quoted five-year overall survival reported at 10–15% [10]. With earlier diagnosis, the treatment options increase, and as more countries employ screening practices, HCC is being detected at earlier stages, and it is likely that this trend will continue. 

There are a number of risk factors for HCC, the most common underlying liver disease of viral aetiology, namely hepatitis B and hepatitis C, but also alcoholic and non-alcoholic cirrhosis. In Asia, the majority of the cases of HCC are secondary to the high prevalence of the hepatitis B virus. Outside of Asia, in the West, Hepatitis C virus (HCV) and alcohol-related cirrhosis are the main contributors to the rising number of cases of HCC [11]. The global burden of HCC tends to follow the burden of viral disease, showing higher documented cases in Asia. Whilst the number of cases in Asia remains high, it is decreasing, whereas in the West, the incidence of liver cancer and specifically HCC, is rising. Dasgupta et al. performed a systematic review and meta-analysis in 2020 of published trends in the incidence of adult liver cancers and histological types worldwide from 31 included studies, using study-specific estimates of the annual percentage change (APC) in incidence rates with 95% confidence intervals (CI). On subgroup analysis, an increasing trend for HCC was noted (APC +3.6, 95% CI +2.9, +4.4) in Europe, North America and Australia [12]. 

There are a number of different staging systems utilised, but the most common is the BCLC staging system, staging patients from very early stage (0) to terminal stage (D) disease, endorsed by EASL and AASLD [13]. This system combines liver biochemical parameters, radiological imaging and performance status with treatment guidelines [Child-Pugh, Model for End-Stage Liver Disease (MELD), Albumin-Bilirubin (ALBI) score, Eastern Cooperative Oncology Group (ECOG) Performance Scale] [14,15]. The algorithm has recently undergone an update, introducing the concept of treatment stage migration (TSM) [16]. TSM requires valuable input from the multidisciplinary team (MDT) as it seeks to consider non-hepatic factors, such as age, donor availability, and co-morbidities, in order to ‘assign’ patients to a more advanced treatment stage than initially recommended by BCLC staging guidelines [4]. Another recent change to the BLCL is the inclusion of the ALBI score as an HCC-specific update. The ALBI score essentially focuses on albumin and bilirubin as the most important prognostic features of the Child-Pugh scoring system, disregarding features that are historically more vulnerable to subjective assessments, such as ascites and encephalopathy. ALBI has a 3-point grade system, with 3 conferring the worst prognosis [17].

The degree of portal hypertension has long been considered an important factor when assessing liver disease. A hepatic venous pressure gradient (HVPG) of >10 mmHg has been linked to a higher rate of complications in the postoperative period and reduced long-term survival rates [18,19]. 

There is an emphasis on shifting the sole focus from tumour burden and liver function alone to the inclusion of factors specific to each individual patient, such as their medical profile and degree of symptoms. 

## 3. Non-Ablative Treatment Options 

### 3.1. Transplant Surgery

HCC accounts for as much as 90% of liver cancer diagnoses [7]. As many as 20–40% of worldwide liver transplants are for the treatment of HCC [20]. OLT is the recognised optimal treatment for patients with the low-volume unresectable disease [21]. Donor organ shortage is a limiting factor to OLT worldwide. 

In current guidance, priority on the waiting list is made for patients with HCC who are on the waiting list for at least six months and are within the Milan Criteria (i.e., one lesion up to 5 cm or up to 3 lesions ≤ 3 cm with no features of macrovascular invasion or extrahepatic disease) [22]. Notably, in recent years, a number of centres have adopted criteria beyond the standard Milan criteria, such as those of the University of California, San Francisco (UCSF). The latter proposed a modest expansion of the Milan criteria with the inclusion of patients with a solitary tumour up to 6.5 cm or up to 3 nodules with the largest lesion up to 4.5 cm and a total tumour diameter up to 8 cm. They showed excellent performance without adverse impact on 5-year survival [23]. The UCSF criteria were further validated independently on either explant pathology or imaging, suggesting that they can predict survival as accurately as the Milan criteria and could therefore serve as selection criteria for liver transplant [24]. A systematic review and meta-analysis from 2021 concluded that the Milan and UCSF criteria confer equivalent survival rates [25].

OLT is the definitive treatment for HCC as it includes the removal of the cirrhotic environment reducing the chance of de novo lesions, while it also removes any undetected microscopic lesions in the diseased liver [26]. 10-year recurrence-free survival (RFS) after resection is 22–25% in comparison with a 10-year RFS after transplant of 50–70% [27,28]. 

Centres must have robust follow-up surveillance programmes involving repeated imaging and serial tumour markers, most commonly alpha-fetoprotein (AFP). Fernandez-Sevilla et al. found that the majority of recurrence was found to be within the first two years but can vary, highlighting that extrahepatic recurrence was not uncommon [29]. 

Whilst direct comparison through clinical trials is difficult in transplant vs. non-transplant patients due to the significant differences in patient cohorts and small geographical variations, the generally accepted consensus is that patients who have advanced cirrhosis, portal hypertension, as well as hepatic decompensation are recommended for transplantation and those without cirrhosis or with Child-Pugh A cirrhosis are recommended for resection [27,30]. 

Crocetti et al. found that up to 10% of patients awaiting transplants demonstrate progressive features that move them outside of the transplant eligibility group [31]. Updated BCLC guidance supports the use of ‘bridging therapies’ to contain tumour burden until transplant or, in some cases, lower tumour burden and effectively “downstage to transplant” [4]. As such, it is currently common practice to employ locoregional, surgical, or systemic therapies in pursuit of this concept [4,32,33].

Furthermore, to decrease the heavy dropout on the transplant waiting list, but also to expand the donor pool, various surgical techniques and advances have been successfully engaged, including living-donor liver transplantation, the use of split liver grafts and the use of marginal livers [20,34,35,36,37]. These have been shown to be safe and confer good outcomes in patients with HCC, which has prompted their increasing utilisation worldwide. [20,35,36,37].

### 3.2. Resection

Resection is recommended for patients with stage 0 and stage A disease. These patients should demonstrate preserved liver function, PS 0 and, as described above, have either a solitary nodule or less than three nodules up to 3 cm. Patients with non-cirrhotic livers are the ideal cohort for surgical resection. The lack of fibrosis and associated inflammatory changes means that these patients are able to undergo more complex and, in some cases, larger resections, including hemihepatectomies or extended hepatectomies. This cohort demonstrates more favourable postoperative outcomes of major resection with morbidity and mortality rates of 33% and 4%, respectively [38,39]. Comorbidities can, however, affect these rates and therefore, a thorough work-up is required; imaging supplemented by a biopsy to assess the integrity of the underlying liver parenchyma prior to intervention in patients with non-cirrhotic livers remains crucial [40]. Active hepatitis, steatosis (seen in NAFLD), and metabolic syndrome are all associated with higher rates of postoperative complications [41,42]. Cirrhosis is associated with an increased risk of major hepatectomy [40]. Hence, in cirrhotic patients, apart from the existing comorbidities, it is of paramount importance to take into consideration a combination of parameters, including Child–Pugh and MELD score, liver stiffness measurement (LSM) by transient elastography, which indicates a significant risk of postoperative liver failure when calculated above 12–14 kPa, assessment for clinically significant portal hypertension (HVPG of >10 mmHg or presence of varices), tumour burden, as well as the relationship with anatomical structures and the volume of the future liver remnant (FLR) [40]. It has been documented that an FLR of at least 40% of the total liver volume is required in cirrhotic patients to undergo major liver resection with a lower risk of postoperative liver failure, while the respective percentage in the absence of cirrhosis has been estimated at 25–30% [40].

Overall, as the burden of HCC cases increases, there is a reflected increase in the associated accepted indications for liver resection allowing for the inclusion of large tumours [26].

In patients with nodules not amenable to ablation due to the associated risk of adjacent organ damage or unsuitable location of the nodule, laparoscopic/robotic resection has provided an option where feasible, being less invasive and, thus, more suitable in patients who would otherwise be suboptimal candidates for an open resection [4]. The current updated guidance requires multifactorial evaluation, but resection remains the recommendation for stage 0 and stage A patients. These patients will typically have a solitary nodule without macrovascular invasion or evidence of extrahepatic disease and preserved liver function with Child-Pugh A. Other factors considered are a MELD score <10 and a sufficient FLR, calculated on imaging using volumetric software [4,28]. 

Even within the recommendations, one still has to take into consideration tumour burden and the location, as well as the risks associated with surgery in comparison to more minimally invasive techniques, such as ablation. Studies comparing RFA and surgical resection show reduced morbidity and mortality in patients undergoing RFA. A randomised controlled trial from 2014 of 120 patients with small HCC (≤3 cm) who underwent either percutaneous RFA or liver resection found the starkest differences with morbidity of 5% vs. 27.5%, respectively [43]. In a more recent prospective randomised study of patients with newly diagnosed solitary HCC, there was an associated morbidity of 37.9% in the liver resection group vs. 26.5% in the RFA group, treating tumours of ≥2 cm but ≤4 cm [44]. In HCC of <3 cm ablation provides a competitive alternative treatment after taking into consideration the location of nodules [45,46,47].

The rate of recurrence post-resection is also commonly high, largely considered to be due to established undetected microscopic disease [48,49]. However, currently, there is no global consensus on adjuvant therapy use in HCC management [7]. Microvascular invasion and satellites are known predictors of recurrence, and the presence of these factors may be used to place a patient in consideration for OLT [7].

Another role for resection is in the treatment of recurrent disease, including after OLT. This has been shown to be superior to systemic therapy as demonstrated by a 96-patient study from the University of Pennsylvania, where patients who underwent ablation or resection for recurrent HCC post-OLT had median survival rates of 33 months for resection, 21 months for ablation and 7.7 months for systemic therapy [50]. 

### 3.3. TACE

The intermediate stage (BCLC B) is a heterogeneous group that has been further subdivided into three subgroups in the latest update. BCLC B is defined as having multifocal HCC with preserved liver function, no vascular invasion or extra-hepatic spread and without cancer-related symptoms. The update subgroup one includes patients who could be candidates for OLT if able to meet extended Milan criteria (tumour size and AFP levels). Patients in the second subgroup are beyond the extended criteria for OLT but have adequate liver function with preservation of the portal flow, and this is the group that is a candidate for TACE. In the third group, the patients have the extensive disease but relatively preserved liver function (PS1–2) and are recommended for systemic therapy [51]. 

Elsewhere in the BCLC update, TACE is recommended in stages 0, A and B with single lesions <8 cm who have either failed other treatments or in whom other treatments are not feasible [4]. However, outcomes for large tumours (8–10 cm) remain relatively poor. It is difficult to elucidate whether this is due to the potential portal venous flow disruption; however, what is known is that larger lesions are typically associated with symptoms, which places patients in the BCLC C category (PS1), and this cohort has poorer survival on comparison to asymptomatic cohorts [4]. The degree of symptoms associated with such large lesions is already linked to poor outcomes. 

### 3.4. Chemotherapy/Immunotherapy

The most recent advances in HCC management have occurred in relation to systemic therapy. Following the results of the IMBRAVE-150 trial, immunotherapy with atezolizumab and bevacizumab is currently used in conjunction as the first-line treatment in patients with advanced-stage (BCLC-C) HCC [52]. Patients in this group will have evidence of vascular invasion with the extrahepatic spread but preserved liver function (compensated Child-Pugh A in underlying cirrhosis) and still relatively fit (PS < 2). The combination of atezolizumab and bevacizumab has been shown to have improved survival in comparison to previously recommended sorafenib [52,53]. In order to benefit from the combination, patients must be at low risk for bleeding and have no vascular disorders, autoimmune disorders or previous organ transplantation [54]. 

More recently, in the phase 3 HIMALAYA trial (NCT03298451), the dual immunotherapy regime combination of durvalumab and tremelimumab has demonstrated an improvement vs. sorafenib as frontline therapy in patients with unresectable HCC with an OS of 16.4 months vs. 13.8 months in sorafenib therapy alone [55,56]. 

In patients who are not deemed suitable candidates for the two combinations, sorafenib or lenvatinib can still be considered. This is an area where prospective studies are expected to significantly aid in fine-tuning the indications for each treatment and offer the best survival benefit. Several ongoing trials aim to glean further data to aid in treatment stratification [7]. 

Patients who receive chemotherapy commonly have either extra-hepatic disease, demonstrate vascular invasion, or have failed to respond to transarterial chemoembolisation techniques [57]. Unfortunately, despite advances in the management of HCC, the outcomes of patients with chemotherapy remain poor [58]. In Japan, where there are high rates of HCC, there are currently two classifications for chemotherapy; systemic chemotherapy for those who have extrahepatic metastatic disease and hepatic artery infusion chemotherapy (HAIC) for those with locally advanced disease. HAIC is shown to reduce tumour size and offer slightly better longer-term survival, but this has yet to be replicated in a randomised controlled trial (RCT) [8]. Similarly, two studies suggested TARE to be as effective as sorafenib in patients with localised hepatic disease. However, these findings have not been corroborated in prospective phase III trials [59,60,61]. 

## 4. Very Early Stage (BCLC 0) 

As this review focuses on ablative techniques, the focus is placed on the groups of patients who have HCC suitable for ablation. The two groups in which ablation is recommended in the BCLC staging system are unresectable cases with BCLC 0 and BCLC A. Very early stage (BCLC 0) HCC accounts for a single lesion ≤2 cm in a patient with preserved liver function and a performance status (PS) of 0. Early stage (A) HCC comprises patients with a single nodule of any size, or <3 nodules of less than 3 cm with preserved liver function. In both of these categories, there must be no evidence of extrahepatic spread and no features of vascular invasion [4]. 

As described above, the stratification of patients into stages allows for the recommendation of appropriate therapy, with patients who have very early or early disease recommended for treatment with curative intent; typically, this includes either surgical resection, OLT or local ablation [4]. 

Currently, ablation is the recommended treatment in patients with BCLC stage 0 disease not suitable for surgical intervention [3]. Otherwise, in those who are surgically fit, where possible, resection is typically recommended. There is extensive literature comparing the outcomes of RFA to hepatic resection, and more recently, more studies emerging on the use of microwave ablation (MWA), which has been shown to be non-inferior to RFA with additional safety benefits [13].

Patients may warrant consideration for transplant in stage 0 if they are considered to have a high recurrence risk, i.e., evidence of microscopic vascular invasion or satellites. However, owing to low organ donor availability and priority policies, typically, recurrence has to first be apparent before this is offered [62]. Similarly, patients with severe hepatic disease/liver decompensation but small lesions may also be considered for OLT. 

Patients who are not suitable for surgical resection and have nodules not amenable to ablation (this may be due to factors such as nodule location or lack of local ablation facilities etc.) can be considered for transarterial chemoembolization (TACE) [63]. There is emerging evidence for stereotactic body radiation, though this requires further studies to elucidate its potential role in HCC management [4]. In cases where patients do not meet the criteria due to factors not associated with HCC, their predicted survival is classified as poor therefore, they are moved to stage D [4].

## 5. Early Stage (BCLC A)

Early stage (BCLC A) is defined as a solitary lesion of any size or up to 3 nodules measuring up to 3 cm without macrovascular invasion or extrahepatic spread in a cohort with preserved liver function [4]. If liver function is not preserved, the patient is classified as Stage D due to poor prognostic factors in the event of no transplant. Whilst surgical management via resection is the recommendation, in some centres ablation may be more readily available. There are numerous studies that support the efficacy of ablation in terms of survival rate when compared with resection. This is however mostly demonstrated in lesions that are up to 2 cm. In larger lesions there are reports of increased recurrence with the rates of complete response falling as lesion size increases [64]. However, several studies have highlighted the advantage in MWA over RFA in this regard, with MWA being effective in lesions up to 4 cm [14,65]. It is important to note that size should not be a limiting factor when considering surgical resection in patients who otherwise have no vascular invasion or extrahepatic disease [4]. 

## 6. Radiofrequency Ablation

**Figure 1:**
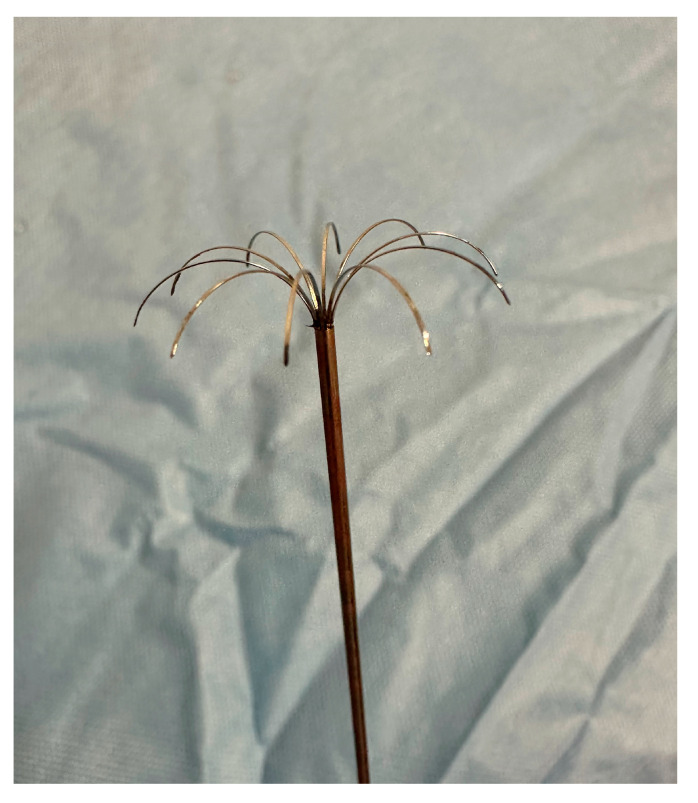
Radiofrequency ablation (RFA) probe with expandable system.

In very early stage (0) and early stage (A) unresectable HCC, thermal ablation is considered the treatment of choice [3]. It has become an accepted treatment for many patients with small volume disease and is the primary treatment for those unable to undergo surgery or liver transplantation. Percutaneous procedures are routinely carried out by Interventional Radiologists with patients typically under general anaesthetic (GA) and with ultrasound (US) or computed tomography (CT) imaging guidance. The main basis of thermoablative techniques, is coagulative necrosis resulting in tissue (tumour) destruction. Carrying out RFA requires 3 main pieces of equipment [66]: an electrode needle (with ablative tynes and thermocouples) (Figure 1), a generator and grounding pads.

A typical RFA system can be single-needle or an expandable system encompassing up to 9 multi-length curved electrodes that can be deployed from an outer needle in an umbrella-like configuration allowing for a wide ablation zone (Figure 1) [3,66]. The electrode is attached to a radiofrequency generator via insulated wires and a current is discharged within the radiofrequency range typically between 375 and 480 kHz, resulting in the motion of ions around the electrode tips causing heat; i.e., heating occurs when the electrical current passes through the ionic tissue medium [3]. The temperatures reached can surpass 60 degrees Celsius resulting in the desired necrosis [3]. The thermocouples act as miniscule thermometers that allow monitoring of temperature [66]. A grounding pad is placed on the patient, usually on the back or thigh, prior to the procedure. Different generator systems can either employ feedback via tissue impedance (tissue resistance) or temperature, which is typically targeted at 90–100 degrees centigrade. A tyne temperature of at least 60 degrees Celsius is required to ensure tumour necrosis. Both of these measures, temperature at the target and tissue resistance, work to reduce the risk of overheating resulting in tissue charring [65]. Once complete the tract is ablated as the electrode is withdrawn to reduce the risk of seeding and bleeding. Typically, a 0.5–1 cm margin is required to ensure acceptable tumour destruction including potential microsatellite inclusion, in a bid to prevent local recurrence [3,67]. 

A recent multicentre study of 140 patients evaluated the efficacy of a ‘no-touch’ RFA technique where there is no direct violation of the tumour during treatment of small lesions (2.5 cm). Technical success was observed in all cases, using either the no-touch approach (*n* = 128) or conversion to tumour puncture (*n* = 12). The no-touch RFA technique had a success rate of 91.4% and was found to be effective and safe for small hepatocellular carcinomas of <2.5 cm, with 1.6% cumulative incidence of local tumour progression at 2 years. Insufficient peritumoural parenchyma was a predictive factor for failure of the no-touch technique [68]. 

## 7. Complications 

Post-ablation syndrome is a phenomenon that is demonstrated in up to a third of patients undergoing ablation. The symptoms are typically self-limiting lasting up to a week and include low-grade pyrexia, delayed pain, malaise, myalgia and nausea [69,70]. Other complications that can arise from ablation include [70]:

- Haemorrhage
- Infection
- Biliary Tract Damage
- Liver failure
- Cutaneous thermal injury
- Hepatic vascular damage

## 8. RFA vs. Surgery 

There are small number studies that purport the efficacy of RFA as primary treatment of small HCC being comparable if not in some cases slightly better than surgical resection. However, in the recent literature surgical resection is consistently associated with a small but demonstrable improvement in overall survival (OS) over RFA [71]. Three RCTs showed improved OS rate with surgical resection [62,72,73]. These findings are corroborated when looking at the most recent study of 188 patients by Li et al., from 2021, when observing the results of treatment of very-early stage HCC; they showed 10-year cumulative OS of 55.2% in the Surgical resection group vs. 31.3% in the RFA group (*p*  <  0.001). No statistical difference was noted in the disease-free survival [71]. There are other benefits to consider however regarding RFA; a RCT in 2019 reported 240 patients where RFA had a better complication rate profile on comparison with resection (7.3% vs. 22.4%, *p*  =  0.001) [62]. Another benefit of RFA over surgery to be considered includes shorter hospital stays which has important consequences for resources and finances in already stretched healthcare systems [72,74]. 

## 9. Microwave Ablation 

Microwave ablation is arguably the more popular ablative technique in use. MWA causes tissue destruction by heating tissues in an applied oscillating electromagnetic field (this is typically between 900–2500 MHz). This specifically works well in polar molecules e.g., H_2_O. As both solid organs and tumours have high percentages of water they respond well to this mode of heating. The system comprises a generator, a power distributor and antennas. An antenna inserted to the target tissue allows the microwave energy to heat the tissue that surrounds it. MWA differs from RFA in that it can heat multiple types of tissue not reliant on needing high electrical conductivity; it is therefore not limited by low thermal conductivity or high impedance tissues [75]. The additional features, such as grounding pads are also not needed in MWA. 

**Figure 2:**
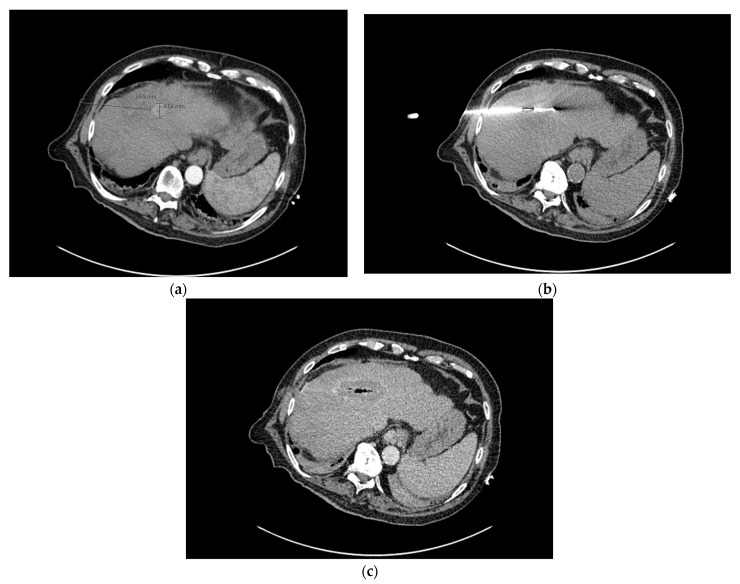
(**a**) Planning CT for microwave ablation of solitary hepatocellular carcinoma (Archive of Dr Fotiadis, The Royal Marsden Hospital). (**b**) Microwave ablation of solitary hepatocellular carcinoma (Archive of Dr Fotiadis, The Royal Marsden Hospital). (**c**) Post-ablation CT (Archive of Dr Fotiadis, The Royal Marsden Hospital).

An additional benefit of MWA is the use of multiple microwave antennas which allows the operator to either ablate multiple tumours synchronously or make use of the thermal synergy with the antennas placed close together to treat a lesion effectively. This subsequently allows the treatment of larger lesions (>3 cm) and creation of larger ablation zones in a more efficient manner [76]. Figure 2a–c shows the CT axial images of the planning and subsequent ablation zone in the treatment of a solitary HCC. 

The use in larger lesions is particularly advantageous given that this has been a problem in RFA, where studies show an unfavourable risk of local tumour progression [77]. MWA has been shown to be the better option when considering ablation in a patient with HCC up to 4 cm [14,65,78]. 

Ablative techniques, mainly RFA and MWA, can be commonly performed both percutaneously and via minimally invasive access. A retrospective analysis of 91 patients over a 5-year period found that the technical approach, when comparing laparoscopic with percutaneous access, did not significantly affect tumour-free survival. It did, however, show that fewer complications were reported after percutaneous ablation in comparison with laparoscopic ablation (14.3% vs. 3.2%, *p*= 0.049) [79]. Laparoscopic ablation had the advantage, however, in the treatment of subcapsular tumours, where it has been shown to demonstrate greater reliability, with average higher energy delivered over tumour size and reduced local tumour progression (7.7% vs. 21.1%) compared to percutaneous MWA [48]. In recent years, advances in stereotactic and robotic thermal ablation of liver malignancies, including a very safe and precise tumour targeting, have resulted in enhanced primary treatment efficacy, as demonstrated in a recent meta-analysis [80]. In accordance, the uptake of robotic access is increasing and is expected to play a more prominent role in the future.

## 10. MWA vs. RFA 

Suitability is another factor to be considered, as the location of the lesions in relation to major vessels has a direct impact on treatment outcomes. Incomplete ablation or recurrent disease can be demonstrated in lesions close to major vasculature. The portal and hepatic veins allow the heat applied to dissipate, resulting in a “heat sink effect”, thus lowering the overall effect and leaving the risk of incomplete ablation or recurrent disease [81]. The heat sink effect is also pathogenetically responsible for the majority of related complications. A study by Tateishi et al. of 1000 treatments of RFA to 2140 HCC nodules in 664 patients identified up to 40 different potential major complications and 17 minor complications, including haemorrhage, perforation, fistulation and seeding [82]. However, the complication rates were low (4% per treatment/1.9% per session for major complications, 1.7% per treatment/0.82% per session for minor complications), accounting for a safe procedure [82].

MWA shares similarly low complication rates alongside RFA. The pathogenesis behind the complications is the same, resulting from heat damage, as well as haemorrhage, infection and abscess [79,83]. MWA also has an emerging role in the prevention of tumour progression in cases where patients are expected to have a waiting time for OLT exceeding 6 months as this carries a risk of progression to factors that rule the patient out of OLT suitability criteria. Transarterial radioembolization (TARE) is also emerging as a treatment option in this category [4].

## 11. Cryotherapy Ablation

Cryotherapy has emerged as a promising local ablation technique, which causes necrosis of tissue by using temperatures as low as <−20 degrees Celsius [84]. Typically using a probe with Argon or Helium gas, ice ball formation and freezing occur by the Joule-Thomson effect [84].

A RCT with 360 treatment-naïve Child-Pugh A or B cirrhotic patients with one or two HCCs  ≤  4 cm without metastatic disease, compared percutaneous cryoablation with RFA [85]. Local tumor progression rates at 1, 2, and 3 years were significantly lower in the cryoablation group, namely 3%, 7%, and 7% vs. 9%, 11%, and 11% for RFA, respectively (*p*  =  0.043). Specifically for HCCs >3 cm, the local tumor progression rate was significantly lower in the cryoablation group, i.e., 7.7% vs. 18.2% (*p*  =  0.041). OS and tumour-free survival were not different in the two groups, while major complications were also similar, i.e., 3.9% vs. 3.3% (*p*  =  0.776) [85]. Perhaps what is even more promising is that percutaneous cryoablation has shown advantages in treating liver tumours including HCC at high-risk locations, particularly those adjacent to various organs [86,87]. 

## 12. Irreversible Electroporation

Irreversible electroporation (IRE) is a relatively new non-thermal ablative procedure, where an electric pulse between two electrodes results in pore formation within the lipid bilayer of a cell membrane, causing subsequent cell death [48]. A unique feature IRE has over the aforementioned ablative techniques is the ability to preserve adjacent important structures, including vascular and biliary structures, as it does not disrupt the extracellular matrix [84,88]. This makes IRE an exciting prospect for the ablative therapy to lesions previously deemed unsuitable due to proximity to structures, such as bile ducts, as well as being less susceptible to the heat-sink effect. Furthermore, IRE causes less damage to surrounding hepatic parenchyma compared to thermoablative techniques [84]. Literature on the application of IRE in the management of HCC is still relatively limited, even more in comparison with other ablative modalities.

A prospective study from 2016 of 25 patients with 48 tumours with a median tumor size of 4.6 cm, 22 of which were HCCs, all treated with IRE, reported local recurrence rates to be significantly associated with tumor size, being 9.7% for lesions <5 cm and 64.7% for those >5 cm [89]. In a study of 55 cirrhotic patients with Child-Pugh B disease treated with either MWA (*n* = 25) or IRE (*n* = 30) Bhutiani et al. found that patients undergoing IRE had shorter length of stay (*p* = 0.05) and readmission rates (*p* = 0.03). Of note, the vast majority of HCCs treated with IRE were close to vascular structures, while such lesions were a small minority in the MWA group. Complication rates were 27% in the IRE group vs. 76% in the MWA group. Treatment success at 90 days was 100% for both modalities. The 180-day success rate was 97% for IRE vs. 100% for MWA (*p* = 0.37) [90]. A recent single-centre propensity-matched retrospective study of 190 HCC ablations comparing IRE and RFA found no significant difference in local recurrence-free survival, while no major complications or deaths were observed in either group. The authors concluded that IRE should be considered as a treatment option in HCC cases before stage-migration to non-curative therapies [91].

## 13. Discussion 

Most studies reviewed either compared the most commonly used ablative techniques, i.e., microwave and radiofrequency, or compared one ablative technique, most commonly RFA, to surgical intervention. There were overall mixed outcomes. As already well established in the literature, surgical resection is superior to ablation in patients who are fit for surgery [72]. What is demonstrated in several studies however is that ablation is at least non-inferior to surgical intervention in patients who cannot otherwise undergo surgery and in some studies non-inferior to resection; hence, providing a valuable treatment option for these patients and those who otherwise chose not to undergo surgery. In addition, there is the decreased complication profile that arises from ablation techniques as they require shorter treatment duration, cause less blood loss and result in shorter length of hospital stay. These benefits should be underlined especially in the context of an overall trend of increasing cases of HCC detected in the West [48].

Regarding MWA and RFA, a large meta-analysis by Huo et al. in 2015, of 2062 patients treated for hepatic lesions, showed comparable 1- and 5-year OS, disease free survival, recurrence rates and adverse events [92]. MWA is the predominant ablation technique in use and the meta-analysis of Glassberg et al. of 28 randomised and observational studies showed the rate of local tumour progression to be 30% lower in MWA, in the absence of significant differences with RFA relating to efficacy and safety outcomes [93].

Furthermore, MWA is consistently reported in several studies to have an advantage in reduction of procedural time, overall hospital stay and subsequently, one would conclude, the overall cost. Several studies reported a consistently reduced treatment time in MWA over RFA (6–30 min vs. 12–72 min, *p* = 0.001) [81,90]. This is likely a promising advantage in reducing the overall complication rate. MWA is also reported to have a reduced risk of burns due to not requiring grounding pads as in RFA [65]. Shorter procedure time is likely to correlate to shorter waiting list times which is of importance as there were studies that showed disease progression on patients awaiting surgical treatment, especially the long waiting lists for transplant [94].

## 14. Future Research

Further studies specifically comparing outcomes according to sizes of lesions are an area of future research, as there are presently mixed results. Resection is shown to be superior to ablation in patients with recurrence in lesions over >3.5 cm showing improved OS and RFS, however in patients with small, <3 cm disease, Kudo et al. found no significant difference in OS and RFS between resection and ablation [73]. MWA was shown to be repeatedly superior to RFA in the management of large lesions >3.5 cm with results showing a reduced local tumour progression rate [95,96], however, the meta-analysis by Glassberg et al. found this superiority of MWA over RFA already reflected in smaller tumours of over 2.5 cm [93]. 

In the case of smaller lesions advancing techniques with artificial intelligence, such as robotic assistance to allow greater accuracy is an exciting prospect and still requires further formal study to glean clear data in the actual statistical outcome and degree of improvement. Similarly, further studies into the no-touch RFA technique and the potential outcomes in terms of local tumour progression and overall survival may impact ablative approaches for small tumours. 

There is strong evidence in the literature that both MWA and RFA provide good locoregional treatment options for patients with stage BCLC 0 and A (with a diameter up to 3 cm). In larger tumours up to 5 cm TACE and ablation in combination as opposed to ablation alone can provide superior results. 

The updated BCLC guidance introduces the concept of TSM, upgrading patient’s treatment options, as well as a down-staging strategy with a role for TACE and TARE in single lesions <8 cm. This concept is supported by the findings of the LEGACY trial, a multicenter, single-arm, retrospective study of radioembolization, which showed favourable response rates and prolonged duration of response in the treatment of unresectable, solitary HCC ≤ 8 cm [97]. The addition of the clinical decision-making component means that treatment options can now factor in local resources, as well as having a more personalised approach to the individual patient.

Further studies, in particular prospective trials, are required to investigate the role of non-thermal ablation techniques, such as IRE and laser, as well as Cryoablation, as there is currently little literature comparing these methods to warrant inclusion in recommendation guidelines. 

What does remain prevalent in the 2022 BCLC update is that the management of HCC remains complex and will continue to rely on the MDT in pursuit of a multifactorial approach to provide personalised decision-making and optimise results for each patient. With emerging therapies, the complexity of subgrouping, indications and treatment is likely to increase. Table 1 summarises recent studies comparing RFA and resection, while Table 2 summarises recent studies comparing MWA and RFA

## Figures and Tables

**Table 1:** Summary of recent studies comparing Radiofrequency ablation (RFA) and Resection.

RFA vs. Resection	Number of Patients	Number of Lesions Size of Lesions	Overall Survival (%)	Disease Free Survival (%)	Recurrence	Complications
Pompili et al., 2013 [98]	544	<3 cm single	4 y = 66.2 vs. 74.4 (*p* = 0.353)	ND	4 y = 57.1% vs. 56% (*p* = 0.765)	2% vs. 4.5% (*p* = 0.101)
NG et al., 2017 [72]	218	<3 cm <3	1 y = 95.4 3 y = 82.3 5 y = 66.4 10 y = 41.8 vs. 1 y = 94.5 3 y = 80.6 5 y = 66.5 10 y = 47.6 (*p* = 0.531)	1 y = 70.6 3 y = 46.6 5 y = 33.6 10 y = 18.6 vs. 1 y = 74.1 3 y = 50.9 5 y = 41.5 10 y = 31.9 (*p* = 0.072)	71.3% vs. 81.7%	ND
Lee et al., 2018 [44]	63	<4 cm	5 y = 86.2 vs. 83.4 (*p* = 0.812)	3 y = 44.1 5 y = 31.2 vs. 3 y = 66.7 5 y = 44.4 (*p* = 0.071)	70.6% vs. 51.7%	37.9% vs. 26.5%, (*p* = 0.330)
Xia et al., 2019 [62]	240	Solitary <5 cm or >1 but <3 nodules of <3 cm	1 y = 87.5 3 y = 52.5 5 y = 38.5 vs. 1 y = 92.5 3 y = 65.8 5 y = 43.6 (*p* = 0.17)	1 y = 85 3 y = 52.4 5 y = 36.2 vs. 1 y = 74.2 3 y = 41.7 5 y = 30.2 (*p* = 0.09)	37.8% vs. 21.7% (*p*= 0.04)	7.3% vs. 22.4% (*p*= 0.001)
Kudo et al., 2021 [73]	302	<3 cm <3	5 y = 70.4 vs. 74.6 (*p* = 0.828)	5 y = 50.5 vs. 54.7 (*p* = 0.498)	ND	ND
Li et al., 2021 [71]	188	<2 cm	1 y = 91.7 3 y = 72.8 5 y = 56.7 10 y = 31.3 vs. 1 y = 99 3 y = 87.6 5 y = 80 10 y = 55.2	1 y = 86.6 3 y = 59.8 5 y = 49.8 10 y = 32.6 vs. 1 y = 90.2 3 y = 72 5 y = 59.3 10 y = 45.9	ND	ND

1 y—1-year; 3 y—3-year; 4 y—4-year; 5 y—5-year; 10 y—10-year; ND—Not declared.

**Table 2:** Summary of recent studies comparing Microwave ablation (MWA) and RFA.

MWA vs. RFA	Number of Patients	Number and Size of HCC	Overall Survival (%)	Disease Free Survival (%)	Incidence of Local Recurrence (%)	Complications %	Technique Effectiveness
Abdelaziz et al., 2014 [99]	111	<3 <5 cm	2 y = 62 vs. 47.4 *p* = 0.49	ND	3.9 vs. 13.5 *p* = 0.04	3.2 vs. 11.1	ND
Santambrogio et al., 2017 [100]	154	Single >5 cm or 2–3 lesions <3 cm	5 y = 37 vs. 50 *p* = 0.185	5-year= 12% vs. 19% *p* = 0.434	Local tumour progression 8.3 vs. 21.2 *p* = 0.034	2 vs. 1	ND
Xu et al., 2017 [45]	460	Single lesion <5 cm or 3 nodules <3 cm	1 y = 99.3 3 y = 90.4 5 y = 78.3 vs. 1 y = 98.7 3 y = 86.8 5 y = 73.3 *p* = 0.331	1 y = 94.4 3 y = 71.8 5 y = 46.9 vs. 1 y = 89.9 3 y = 67.3 5 y = 54.9 *p* = 0.309	9.6 vs. 10.1 *p* = 0.883	0.7 vs. 0.6 *p* = 0.691	98.3 (295/301) vs. 98.1 (156/159) *p* = 0.860
Yu et al., 2017 [78]	403	<3 <5 cm	1 y = 96.4 3 y = 81.9 5 y = 67.3 vs. 1 y = 95.9 3 y = 81.4 5 y = 72.7 *p* = 0.91	1 y = 94 3 y = 70.6 5 y = 36.7 vs. 1 y = 93.8 3 y = 66 5 y = 24.1 *p* = 0.07	ND	3.4 vs. 2.5 *p* = 0.59 Needle seeding GI bleeding Bulk pleural effusion	99.6 vs. 98.9 *p* = 0.95
Vietti Violi et al., 2018 [67]	144	<3 <4 cm	ND	ND	2 y = 6% vs. 12% *p* = 0.27	2.8 vs. 4.1	
Kamal et al., 2019 [101]	56	<3 <5 cm	ND	1 y = 92.3 vs. 90.9 *p* = 0.932	1 y = 9.1 vs. 9.1 *p* = 1.00	ND	ND
Chong et al., 2020 [102]	93	<3 <5 cm	1 y = 97.9 3 y = 67.1 5 y = 42.8 vs. 1 y = 93.5 3 y = 72.7 5 y = 56.7 *p* = 0.899	1 y = 51.1 3 y = 24.1 vs. 1 y = 58.7 3 y = 22.7 *p* = 0.912	ND	ND	ND

HCC—hepatocellular carcinoma; 1 y—1-year; 2 y—2-year; 3 y—3-year; 5 y—5-year; ND—Not declared; GI—gastrointestinal.

## Data Availability

Not applicable.
